# The Flux of Select NSAIDs through Silicone Membranes from Mineral Oil

**DOI:** 10.3390/pharmaceutics6030354

**Published:** 2014-07-02

**Authors:** Paul S. Mertz, Kenneth B. Sloan

**Affiliations:** Department of Medicinal Chemistry, University of Florida, P.O. Box 100485, Gainesville, FL 32610, USA; E-Mail: pmertz@ufl.edu

**Keywords:** solubility in mineral oil, solubility in water, silicone membrane surrogate, human skin *in vitro*, Roberts–Sloan equation

## Abstract

Here we report the experimental log maximum fluxes of *n* = 9 non-steroidal anti-inflammatory drugs (NSAID) through silicone membranes from the lipid mineral oil (experimental (Exp.) log *J*_MPMO_) and correlate those Exp. log *J*_MPMO_ values with their experimental log maximum fluxes through human skin *in vivo* from mineral oil (Exp. log *J*_MHMO_). The correlation was only fair (*r*^2^ = 0.647) for *n* = 9 but improved dramatically if Nabumetone was removed from the correlation (*n* = 8, *r*^2^ = 0.858). Non-linear regression of the *n* = 8 Exp. log *J*_MPMO_ values as the dependent variable against their log solubilities in mineral oil (log *S*_MO_) and in pH 7.4 or 1.0 buffers (log *S*_7.4_ or *S*_1.0_, respectively), and their molecular weights as independent variables in the Roberts–Sloan (RS) equation gave a new set of coefficients for the independent variables in RS. Those coefficients have been used to calculate log *J*_MPMO_ values which have been correlated with the Exp. log *J*_MPMO_ values to give *r*^2^ = 0.911 if log *S*_7.4_ and *r*^2^ = 0.896 if log *S*_1.0_ were used as aqueous phases. Thus, silicone membranes appear to be good surrogates for predicting flux through human skin if the vehicle is a lipid such as mineral oil.

## 1. Introduction

In the presently available literature, there is an increasing body of information examining whether the flux of pharmaceuticals through silicone membranes can serve as a surrogate for flux through human or animal skin [1,2]. The need for this alternative method of predicting transdermal drug delivery arises in part due to the European Union’s ban on topical drug or cosmetic formulations tested on animals [3]. Among the literature on models predicting flux, the Roberts–Sloan (RS) equation (Equation (1)) that proposes that maximum flux (*J*_M_) of a molecule through a membrane can be mathematically predicted when the molecular weight (MW), water solubility (*S*_AQ_), and lipid solubility (*S*_LIPID_) of the permeant are known, has been shown to be quite versatile [4].


log *J*_M_ = *x* + *y* log *S*_LIPID_ + (1 - *y*) log *S*_AQ_ – *z* MW (1)

Not only does the RS equation predict log *J*_M_ when an aqueous vehicle is used, but it can be used to predict log *J*_M_ when a lipid vehicle is used. If an aqueous vehicle is used, it can be shown that Fick’s law Equation (2) can be expanded to Equation (1) as follows:

*J* = (*D*/*L*)( *C*_M1_ - *C*_Mn_)
*J*_M_ = (*D*/*L*)( *S*_M1_ - *C*_Mn_)
*S*_M1_ = (*S*_VEH_)(*K*_M1:VEH_)
*S*_M1_ = (*S*_AQ_)(*K*_M1:AQ_) (2)

where *D* is the diffusion coefficient, *L* is the thickness of the membrane, *C*_M1_ is the concentration of the molecule in the first few layers of the membrane, *C*_Mn_ is the concentration in the last few layers of the membrane, *S*_M1_ is the solubility in the first few layers of the membrane, *S*_VEH_ is the solubility in the vehicle (VEH), *S*_AQ_ is the solubility in water (AQ), *K*_M1:VEH_ is the partition coefficient for the molecule between the first few layers of the membrane M1 and the vehicle and *K*_M1:AQ_ is the partition coefficient where the vehicle is water. If solubility in octanol, *S*_OCT_, is used as a lipid surrogate for *S*_M1_ in *K*_M1:AQ_, (*K*_OCT:AQ_)*^y^*∙c, where c is a constant, can be substituted for *K*_M1:AQ_ to give Equation (3):

*S*_M1_ = (*K*_OCT:AQ_)*^y^*∙c (*S*_AQ_)
log *S*_M1_ = *y* log *S*_OCT_ - *y* log *S*_AQ_ + log c + log *S*_AQ_ log *S*_M1_ = *y* log *S*_OCT_ + (1 - *y*) log *S*_AQ_ + log c (3)

If *D*_0_ exp (–*z* MW) is substituted for *D* in Equation (2), *L* is assumed to be a constant in Equation (2), *C*_Mn_ is assumed to approach zero in Equation (2) and Equation (3) is substituted for *S*_M1_ in Equation (2), collection of log c, log *D*_0_ and log *L* into a new constant x gives Equation (1) where the lipid phase is octanol and the vehicle is water: Equation (4).


log *J*_MAQ_ = x + *y* log *S*_OCT_ + (1 - *y*) log *S*_AQ_ - *z* MW (4)

In order to accommodate a lipid vehicle, *K*_M1:VEH_ in Equation (2) becomes *K*_M1:LIPID_ which can be substituted for by *K*_M1:AQ_/*K*_LIPID:AQ_. Since *K*_M1:AQ_ can be substituted for by (*K*_LIPID:AQ_)^y^∙c, as in Equation (3), *K*_M1:__LIPID_ becomes (*K*_LIPID:AQ_) ^y^·c/*K*_LIPID:AQ_ and *S*_M1_ becomes Equation (5):

*S*_M1_ = [(*K*_LIPID:AQ_)*^y^*∙c/*K*_LIPID:AQ_](*S*_LIPID_)
log *S*_M1_ = *y* log *S*_LIPID_ - *y* log *S*_AQ_ + log *c* - log *S*_LIPID_ + log *S*_AQ_ + log *S*_LIPID_ log *S*_M1_ = *y* log *S*_LIPID_ + (1-*y*) log *S*_AQ_ + log c (5)

Thus log *S*_M1_ has the same form in Equation (5) as in Equation (3) and Equation (4) becomes Equation (6) where any lipid vehicle such as mineral oil, MO, can be used in the RS equation: Equation (1) is the same form as Equation (6) but the coefficients will be different depending on the membrane and the actual vehicle and lipid surrogate for M1 used:

log *J*_MLIPID_ = x + *y* log *S*_LIPID_ + (1 - *y*) log *S*_AQ_ – *z* MW (6)

A recently collected *n* = 70 database of compounds for maximum flux through silicone membrane from water (log *J*_MPAQ_, where “P” stands for a polydimethylsiloxane membrane) and an *n* = 55 database for maximum flux through human skin *in vivo* from water (log *J*_MHAQ_, where “H” stands for a human skin membrane) were each individually fitted to the RS equation with good results [2]. An *n* = 52 subset of log *J*_MPAQ_ values were common to the *n* = 55 log *J*_MHAQ_ database. The correlation of the *n* = 52 log *J*_MPAQ_ data with their corresponding log *J*_MHAQ_ was good and suggests that log *J*_MHAQ_ can be predicted from known log *J*_MPAQ_ data [2].

The literature currently lacks substantial data for maximum flux through human skin and silicone from vehicles other than water that can be used to determine if there is any correlation between fluxes through the two membranes. It is unknown if flux from a non-aqueous vehicle through silicone can predict flux from a non-aqueous vehicle through human skin. An *n* = 30 database of flux of Naltrexone prodrugs through human skin *in vitro* from mineral oil (log *J*_MHMO_) has been collected [5]. However, it would be inconvenient as a first step to determine flux of these same prodrugs through silicone membrane from mineral oil (log *J*_MPMO_) since each compound would need to be independently synthesized first. A much more convenient approach to answer the question above would be to determine the experimental log *J*_MPMO_ for commercially available pharmaceuticals that have already been studied in human skin. The log *J*_MHMO_ (*in vivo*) for an *n* = 10 group of non-steroidal anti-inflammatory drugs (NSAID) has been published [6] and that database was found to give a good fit to the RS equation [7].

The data collected here is for the experimentally determined log *J*_MPMO_ (Exp. log *J*_MPMO_) for *n* = 9 of the NSAIDs utilized in the *in vivo* human skin study [6]. One compound (Tenoxicam) was not utilized due to unavailability. Once the Exp. log *J*_MPMO_ data were obtained, a determination was made of how well Exp. log *J*_MPMO_ correlates with Exp. log *J*_MHMO_. A new set of coefficients from the fit of the Exp. log *J*_MPMO_ data for the *n* = 9 NSAIDs and their corresponding solubilities and MWs to RS was obtained and used to calculate log *J*_MPMO_ (calculated (Calc.) log *J*_MPMO_). The Exp. log *J*_MPMO_ data was also fit to various iterations of the RS equation using coefficients which were previously determined from Exp. log *J*_MPAQ_ or log *J*_MHMO_.

## 2. Materials and Methods

### 2.1. Materials

The Franz diffusion cells (surface area 4.9 cm^2^, receptor phase volume 20 mL) were obtained from Crown Glass (Somerville, NJ, USA) and the water bath was from Fisher Scientific (Pittsburg, PA, USA). Diclofenac, Ibuprofen, Ketoprofen, and Naproxen were purchased from TCI (Tokyo, Japan). Nabumetone, Piroxicam, Diflunisal, and Theophylline were purchased from Sigma-Aldrich (St. Louis, MO, USA). Flufenamic Acid was purchased from Acros Organics (Geel, Belgium). Aspirin was purchased from Eastman Kodak Chemicals (Rochester, NY, USA). Silicone membranes (0.254 mm) were purchased from Pillar Surgical (La Jolla, CA, USA) and all solvents were purchased from Fisher Scientific.

### 2.2. Solubilities

The solubilities of each NSAID in octanol (*S*_OCT_), pH 7.4 phosphate buffer solution (*S*_7.4_), and acidic water (*S*_1.0_) were calculated from Wenkers and Lippold’s partition coefficients in their Table 1 and Table 2 and given here in Table 1 [6]. Experimental solubility in mineral oil from Wenkers and Lippold [6] was converted to units of mM from mg/dL. The values of log *S*_7.4_ were calculated from log *S*_OCT_ − log *K*_OCT:7.4_, the values of log *S*_OCT_ were calculated from log *K*_OCT:MO_ + log *S*_MO_, and the values of log *S*_1.0_ were calculated from log *S*_MO_ − log *K*_MO:1.0_.

**Table 1:** Molecular weights; calculated log partition coefficients; calculated solubilities; flux through human skin *in vivo*; flux through silicone membrane.

No.	Compound	MW	log *K*_OCT:MO_^a,c^	log *K*_MO:1.0_^a,d^	log *S*_OCT_^a,e,h^	log *S*_7.4_^a,f,h^	log *S*_1.0_^a,g,h^	log *J*_MHMO_^a,i^	log *J*_MPMO_^b,i^
1	Diclofenac	296	2.91	1.41	1.89	−0.09	−2.43	−2.89	−1.91
2	Flufenamic Acid	281	1.93	2.74	2.46	0.31	−2.21	−1.86	−0.60
3	Ibuprofen	206	1.15	3.06	3.24	2.02	−0.97	−0.25	0.34
4	Ketoprofen	254	3.54	−0.19	3.24	3.27	−0.11	−2.09	−1.18
5	Naproxen	230	2.84	0.38	2.37	2.02	−0.85	−2.00	−1.46
6	Nabumetone	228	0.57	2.72	1.76	−1.12	−1.53	−1.10	−1.53
7	Piroxicam	331	1.49	0.20	0.75	0.95	−0.94	−2.40	−2.08
8	Aspirin	180	3.96	−2.02	3.14	5.18	1.26	−1.72	−1.18
9	Diflunisal	250	3.36	0.88	2.19	1.61	−2.05	−2.44	−1.69

^a^ From Wenkers and Lippold 1999; ^b^ Measured directly; ^c^ Calculated from log *K*_OCT:1.0_ − log *K*_MO:1.0_; ^d^ Calculated from log *K*_OCT:1.0_ − log *K*_OCT:MO_; ^e^ Calculated from log *K*_OCT:MO_ − log *S*_MO_; ^f^ Calculated from log *S*_OCT_ − log *K*_OCT:7.4_; ^g^ Calculated from log *S*_MO_ − log *K*_MO:1.0_; ^h^ Units of mM; ^i^ Units of µmol cm^−2^ h^−1^.

Experimental solubilities in octanol, *S*_OCT_, for each of the compounds were also measured experimentally in this work according to general procedures previously published [8]. An amount of each NSAID expected to saturate 2 mL of octanol was estimated from the previously calculated solubility values. Suspensions of NSAIDs in excess of the estimated values were made in triplicate and were stirred overnight. Samples of NSAIDs in acetonitrile were made with known concentrations and their absorption data were measured using UV spectroscopy. A plot of absorbance *versus* concentration produced a slope that represented the molar extinction coefficient in units of M^−1^. Using these experimental molar extinction coefficients and the calculated solubility in octanol, dilution factors were calculated to place the saturated solution absorbance values between 2.000 and 3.000. Concentrations of the saturated solutions were then calculated from experimentally measured absorbance values and the dilution factors used. These data were collected to verify the *S*_OCT_ data calculated from Wenkers and Lippold [6]. Because the data were similar (percent variation ranged from 2% to 17%), none of the experimentally determined octanol solubility values were used in calculations or analysis to be consistent with the rest of the Wenkers and Lippold data [6].

### 2.3. Molar Extinction Coefficients in pH 7.1 Phosphate Buffer

Molar extinction coefficients for the compounds in pH 7.1 phosphate buffer, the receptor phase, were determined experimentally. Each compound was initially dissolved in a small volume of acetonitrile (except Diflunisal, which was dissolved in ethanol). These solutions were diluted further with the buffer solution to concentrations that would produce accurate UV absorptions. A plot of absorbance *versus* concentration produced the slope in units of M^−1^. Table 2 contains the λ_max_, molar extinction coefficient, and standard deviation for each compound. There was only one instance of a major variation in this procedure. Aspirin readily hydrolyzed to salicylic acid and acetic acid in the phosphate buffer. Thus, the molar extinction coefficient and the concentrations measured after 24 h were that of salicylic acid.

**Table 2:** λ_max_, molar extinction coefficients in pH 7.1 phosphate buffer, and standard deviations (SD).

Compound	λ_max_ (nm)	ε ± SD (M^−1^)
Diclofenac	276	10,423 ± 74
Flufenamic acid	288	14,075 ± 228
Ibuprofen	265	299 ± 37
Ketoprofen	260	14,791 ± 818
Naproxen	271	5,116 ± 433
Nabumetone	271	6,631 ± 334
Piroxicam	286	9,778 ± 131
Aspirin	295	3,389 ± 96
Diflunisal	305	6,895 ± 1,009

### 2.4. Diffusion Cell Experiments: Determination of log J_MPMO_

Suspensions were prepared by stirring 0.5 g of each NSAID (1.0 g of Ibuprofen) in 10 mL of light mineral oil for 24 h. The experiments were run in triplicate using silicone membranes placed in Franz static diffusion cells that were kept at a constant 32 °C temperature with a circulating water bath according to general procedures previously published [8]. The membranes were kept in contact with a de-ionized water donor phase and pH 7.1 phosphate buffer receptor phase for 3 h to condition the membranes. A 1 mL aliquot of the NSAID suspension was applied to each of the membranes as the donor phase, and after 5 h the receptor phases were changed. The suspensions were left on the membrane for 16 more hours. Samples of the receptor phases were then taken at 21 h and analyzed by UV spectroscopy to determine the most appropriate sampling time to ensure that the sample would produce accurate UV absorption values and the receptor phases would maintain sink conditions. Further samples were taken every 3 h for all compounds except for Flufenamic acid, Nabumetone and Aspirin (2 h), and Piroxicam (4 h) so that at least four samples were taken for each NSAID. Donor phases were changed as needed to maintain suspensions throughout the experiment.

After the first application, membranes were washed 3–4 times with ethanol to remove the suspensions. Methanol was then left in the donor phase to leach out any remaining residue in the membrane. All membranes were leached for 48 h. After leaching, a 1 mL aliquot of 600 mg/9 mL of theophylline in propylene glycol (Th/PG) was applied as the donor phase. Receptor phases were sampled and replaced every 24 h for a total of 3 samples. The flux of Th/PG, log *J*_MPPG_, was compared to previous control flux values to determine if the silicone membranes had been altered by the first applications. Altered membranes would produce higher fluxes than the literature values [8]. All second application fluxes of Th/PG were within the SD of previously reported values. The diffusion cells were dismantled and cleaned after the second application. Membranes were cleaned by soaking in ethanol for 24 h, followed by methanol for 48 h.

Flux values, log *J*_MPMO_ and log *J*_MPPG_, were obtained by plotting cumulative amounts of permeated compound in the receptor phase from 21 h until at least four samples were obtained *versus* time. The slopes of the plots were calculated as µmole per hour (*r*^2^ at least 0.949 for all plots) and divided by the area of the membrane to provide the units of flux, µmol cm^−2^ h^−1^.

### 2.5. Calculations

Linear regression correlations were made between experimental log *J*_MPMO_ and Wenkers and Lippold log *J*_MHMO_. Nonlinear regression analyses of the present data were utilized to determine coefficients for a new Roberts-Sloan equation and were performed using SPSS 9.0.0 (SPSS Inc., Chicago, IL, USA). The SPSS statistical software was not used to calculate the statistical significance of each coefficient individually, but only for regressions that gave a good *r*^2^.

Flux values were also calculated from the coefficients to previously published iterations of the RS equation and these calculated log *J*_MPMO_ were used in linear regressions against experimental log *J*_MPMO_. All plots and linear regressions were performed with Excel 14 (Microsoft Corporation, Redmond, WA, USA) and OriginPro 8.5.1 (OriginLab Corporation, Northampton, MA, USA).

## 3. Results and Discussion

### 3.1. Comparison of Flux through Silicone Membranes and Human Skin in Vivo

**Figure 1:**
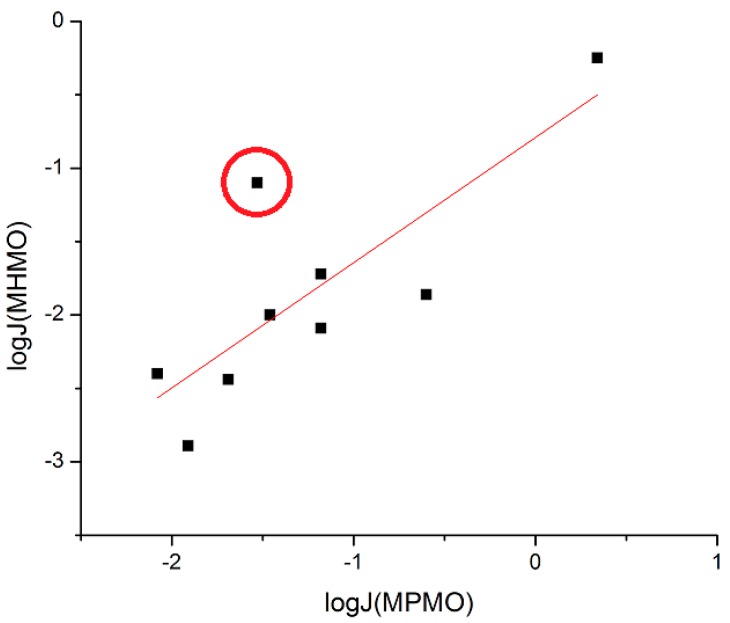
Plot of experimental (Exp.) log *J*_MHMO_ *versus* Exp. log *J*_MPMO_. The circled point is Nabumetone.

The first analysis performed was how well the experimentally determined flux through silicone membrane (log *J*_MPMO_) compared with the human skin *in vivo* (log *J*_MHMO_) dataset published by Wenkers and Lippold [6]. The log *J*_MHMO_ dataset was plotted *versus* log *J*_MPMO_ and a linear regression was performed (Figure 1). It yielded a fair correlation (*r*^2^ = 0.647). A notable outlier in the log *J*_MPMO_ data was Nabumetone. Flux through silicone membranes is typically higher than flux through human skin, as shown in a recent review article on silicone membranes [1]. However, the log *J*_MPMO_ of Nabumetone was notably lower than its log *J*_MHMO_. A major concern with this data was that the Nabumetone data was originally collected in 3 h intervals and it was hypothesized that the receptor phase was no longer under sink conditions (concentration at less than 30% solubility). This saturation of the receptor phase would cause the measured flux to not be the maximum flux, *J*_M_. The Nabumetone diffusion cell was performed again with 2 h collection intervals. Even shorter intervals were preferred but smaller intervals would have made the measured UV absorbance to be too low for accurate measurements. Though these cells produced receptor phases with ≤20% saturation, flux was almost unchanged (log *J*_MPMO_ = −1.53 *versus* −1.55 in the first procedure). These results cannot be explained currently but will be further investigated when more log *J*_MHMO_ data are available for comparison. A new plot and linear regression analysis of the data with Nabumetone removed from the dataset yields a better correlation between human skin *in vivo* and silicone membranes (circled data point in Figure 1 removed. *r*^2^ = 0.858).

### 3.2. Calculation of Roberts–Sloan (RS) Equation Coefficients Derived from Experimental Data

New RS equation coefficients for the present data were estimated using nonlinear regression. In total, eight regressions were performed using the log *J*_MPMO_ data. Regressions were performed using either the *n* = 9 dataset or *n* = 8 (Nabumetone removed). The regressions were performed with each possible combination of log *S*_OCT_ or log *S*_MO_ for lipid solubility and log *S*_7.4_ or log *S*_1.0_ for aqueous solubility.

The first regressions performed utilized log *S*_OCT_ as lipid solubility and log *S*_7.4_ as water solubility in the RS equation. The majority of the literature regarding silicone membranes as surrogates for measuring flux utilize log *S*_OCT_ in the RS equation or in other related equations. It therefore seemed logical to attempt to estimate equation coefficients using the same lipid solubility. For the *n* = 9 NSAID database and log *S*_7.4_ as aqueous solubility, the coefficient estimates were *x* = −4.550 (±1.119), *y* = 1.172 (±0.141), *z* = −0.0033 (±0.0045), and *r*^2^ = 0.558. These calculated coefficients differ from much of the previous available literature. The *y* coefficient is above 1, meaning there is an inverse relationship between log *S*_7.4_ and log *J*_MPMO_. Similarly, a negative *z* coefficient implies a direct relationship with molecular weight and log *J*_MPMO_. A regression with Nabumetone removed yielded similar coefficient estimates (*x* = −4.567, *y* = 1.168, and *z* = −0.0034) with a similar correlation of *r*^2^ = 0.549.

When utilizing log *S*_1.0_ for aqueous solubility and keeping *S*_OCT_ as lipid solubility, regression of the *n* = 9 database provides coefficient estimates of *x* = −4.999 (±1.386), *y* = 1.062 (±0.210), *z* = −0.0048 (±0.0048), and *r*^2^ = 0.457. The peculiarities of the previous regressions are the same for log *S*_AQ_ = log *S*_1.0_. Analysis of the *n* = 8 database in the same manner produces similar coefficients with a similar correlation: *x* = −5.233, *y* = 1.068, *z* = −0.0054, and *r*^2^ = 0.493.

Noting the poor *r*-squared values using log *S*_OCT_, regressions were performed instead using log *S*_MO_ for lipid solubility. The coefficient estimates for the *n* = 9 database using log *S*_7.4_ as *S*_AQ_ were *x* = −2.592 (±0.790), *y* = 0.734 (±0.058), *z* = −0.0039 (±0.0030), and *r*^2^ = 0.803. Use of mineral oil solubility showed a dramatic improvement in the fit of the parameter estimates to the RS equation. However, the *z* coefficient was still notably negative despite the previous literature typically yielding positive values. A regression with the same solubilities but with removal of Nabumetone substantially improves the fit with coefficients of *x* = −1.755 (±0.799), *y* = 0.822 (±0.067), *z* = −0.0015 (±0.0028), and *r*^2^ = 0.882.

Using log *S*_1.0_ for aqueous solubility and *S*_MO_ for lipid solubility, the *n* = 9 database coefficient estimates provided a poor fit with values of *x* = −1.375 (±1.131), *y* = 0.703 (±0.126), *z* = −0.0020 (±0.0045), and *r*^2^ = 0.533. This fit was greatly improved to *r*^2^ = 0.846 with removal of Nabumetone from the regression and coefficient estimations of *x* = −0.712 (±0.735), *y* = 0.823 (±0.067), and *z* = 0.00053 (±0.0029). This set of coefficients is notably different from those produced in other regressions. It is the only set where the *z* coefficient was estimated as a positive value as would be predicted based on prior literature.

In both regressions with good *r*^2^, the *z* coefficient is statistically insignificant (*p* = 0.615 and 0.862, respectively). This issue was previously noted in the analysis of the Wenkers and Lippold human skin *in vivo* data and is likely due to the narrow range of MW compounds used [7]. However, because the *z* coefficient is reasonably similar to previous iterations of the RS equation [1,2,5], log *J*_M_ should retain its dependence on the MW until more flux data can be experimentally determined.

**Figure 2:**
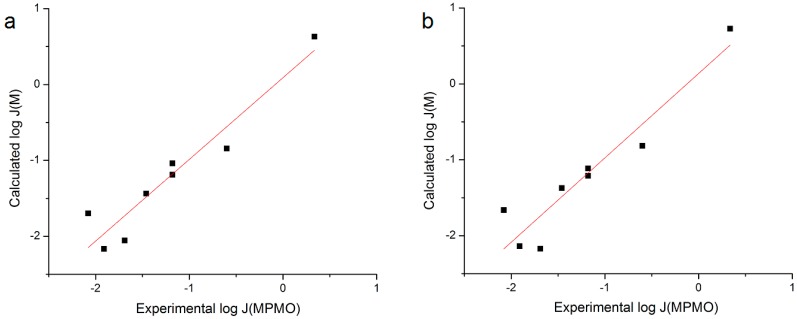
Plot of log *J*_M_ calculated with the coefficients derived from nonlinear regressions performed in this work *versus* *n* = 8 Exp. log *J*_MPMO_. (**a**) Dataset using *n* = 8, *S*_MO_, *S*_7.4_ and (**b**) Dataset using *n* = 8, *S*_MO_, *S*_1.0_.

The regressions with the best fit were obtained from the *n* = 8 dataset using log *S*_MO_ and either log *S*_7.4_ (*r*^2^ = 0.882) or log *S*_1.0_ (*r*^2^ = 0.846). Plots of Calc. log *J*_MPMO_ using the estimated coefficients from the fit of the *n* = 8 dataset to RS where *S*_MO_ was the lipid phase *versus* Exp. log *J*_MPMO_ are provided in Figure 2. These linear plots give good correlations of *r*^2^ = 0.911 (*S*_7.4_) and *r*^2^ = 0.896 (*S*_1.0_).

### 3.3. Comparison of Experimental log J_MPMO_ with Calculated log J_M_ Using S_OCT_ as the Lipid Solubility Term

The final analysis performed was of the linear regression between the experimental log *J*_MPMO_ data and those calculated from the previously published coefficients to the various iterations of the RS equations below. Because there was prior literature for *S*_7.4_ and *S*_1.0_ for the NSAIDs examined [6], calculations of flux were done using both *S*_7.4_ and *S*_1.0_. The variables *x*, *y*, and *z* are all coefficients estimated from previous non-linear regressions on experimentally determined data. Coefficients used in these analyses are reproduced in Table 3.

**Table 3:** RS equation coefficients from previous literature and *S*_LIPID_ utilized.

Reference	*x*	*y*	*z*	*S*_LIPID_
Sloan, *et al.* [1]	−1.607	0.701	0.00492	*S*_OCT_
Prybylski, *et al.* [2] (*n* = 70)	−1.606	0.695	0.00490	*S*_OCT_
Prybylski, *et al.* [2] (*n* = 55)	−3.005	0.654	0.00112	*S*_OCT_
Sloan, *et al.* [5]	−1.823	0.462	0.00153	*S*_MO_
Roberts, *et al.* [7]	−1.459	0.722	0.00013	*S*_MO_

**Figure 3:**
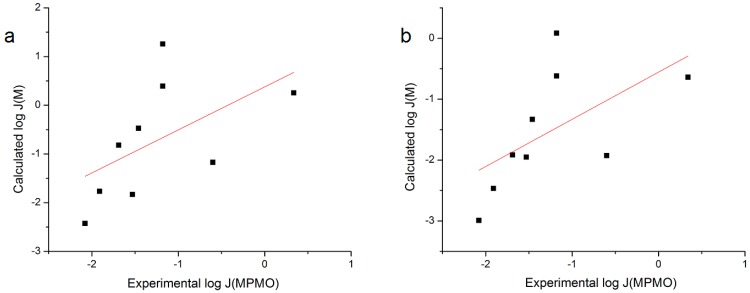
Plot of log *J*_M_ calculated with Sloan, *et al.* surrogate review coefficients [1] *versus* Exp. log *J*_MPMO_ using (**a**) S_7.4_ or (**b**) S_1.0_.

The first iteration of the RS equation (Equation (4)) used was from the Sloan *et al.* review on surrogates for topical delivery in human skin [1]. The coefficients used [1] were calculated from an *n* = 63 database of flux of molecules through silicone membranes from water. A plot and linear regression of calculated log flux values from Equation (4) *versus* experimental log flux for the *n* = 9 NSAIDs was performed and these data are shown in Figure 3. The data yields a poor correlation for each of the regressions performed (*r*^2^ = 0.295 or 0.341 when using *S*_7.4_ or *S*_1.0_, respectively). Removal of Nabumetone from the dataset slightly reduces each *r*^2^ value (0.284 and 0.330, respectively).

A recent paper by Prybylski, *et al.* expanded on the *n* = 63 database with an additional seven substituted phenolic compounds to give an *n* = 70 database [2]. This addition provided an improved fit of the *n* = 70 compounds to the RS equation with a slight change in the coefficient values [2]. A second linear regression of the *n* = 9 NSAIDs was performed here with the altered coefficients [2]. The correlation for each regression performed was still poor (*r*^2^ = 0.292 or 0.337 when using *S*_7.4_ or *S*_1.0_, respectively). Removal of Nabumetone from the dataset once again slightly reduced each *r*^2^ value (0.280 and 0.326, respectively).

**Figure 4:**
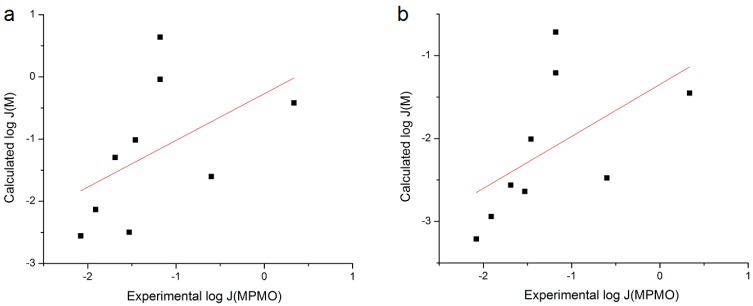
Plot of log *J*_M_ calculated with Prybylski, *et al.* coefficients [2] from the *n* = 55 database *versus* Exp. log *J*_MPMO_ using (**a**) S_7.4_ or (**b**) S_1.0_.

The Prybylski work also expanded the *n* = 48 database of log *J*_MHAQ_ to give an *n* = 55 database. The plot of flux calculated using the coefficients [2] to the fit of RS to the *n* = 55 database *versus* the experimental log *J*_MPMO_ produced a poor correlation, *r*^2^ = 0.249 using *S*_7.4_ or 0.302 using *S*_1.0_, that was further decreased with the removal of Nabumetone from the data (*r*^2^ = 0.241 and 0.289, respectively). These plots are shown in Figure 4.

### 3.4. Comparison of Experimental log J_MPMO_ with Calculated log J_M_ Using S_MO_ as the Lipid Solubility Term

**Figure 5:**
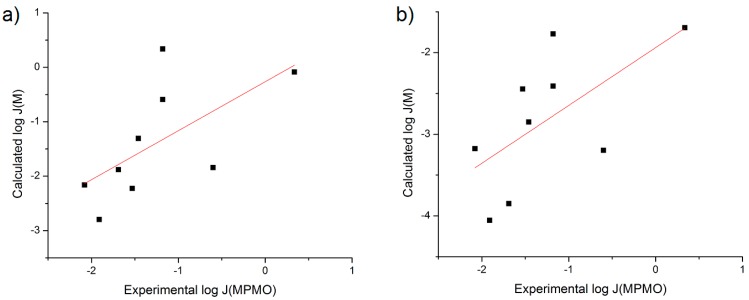
Plot of log *J*_M_ calculated with Stinchcomb *et al.* coefficients [5] *versus* Exp. log *J*_MPMO_ using (**a**) *S*_7.4_ or (**b**) *S*_1.0_.

Another set of coefficients utilized was obtained from an analysis of pooled *n* = 30 of Stinchcomb *et al.* data on the flux of Naltrexone prodrugs through human skin *in vitro* from mineral oil [5]. Flux of NSAIDs was calculated from coefficients to the fit of RS to the *n* = 30 database using Equation (7) and plotted *versus* experimental log *J*_MPMO_ (Figure 5). The correlation between these data was poor (*r*^2^ = 0.393) when using *S*_7.4_ and was essentially unchanged using *S*_1.0_ (*r*^2^ = 0.398).


log *J*_MAQ_ = x + *y* log *S*_MO_ + (1 - *y*) log *S*_AQ_ – *z* MW (7)

**Figure 6:**
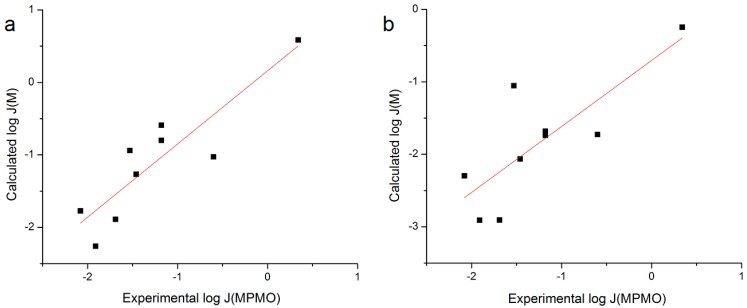
Plot of log *J*_M_ calculated with the coefficients from the Roberts *et al.* analysis of Wenkers and Lippold [7] *versus* Exp. log *J*_MPMO_ using (**a**) *S*_7.4_ or (**b**) *S*_1.0_.

A final regression was performed utilizing coefficients derived from an analysis of data from the Wenkers and Lippold human skin *in vivo* experiment [7] fit to Equation (7). There was a reasonable correlation between the log *J*_MPMO_ values for the *n* = 9 NSAIDs calculated from Equation (7) and experimental log *J*_MPMO_ obtained here: use of *S*_7.4_ yielded *r*^2^ = 0.792 and use of *S*_1.0_ yielded *r*^2^ = 0.635 (Figure 6). These correlations were improved with the removal of Nabumetone from the dataset (*r*^2^ = 0.831 and 0.832, respectively).

## 4. Conclusions

Comparison of flux for the *n* = 9 NSAID compounds through silicone and through human skin *in vivo* yields a fair correlation that is vastly improved with the removal of the outlier Nabumetone from the dataset. Further investigation of this anomaly is warranted*.* Calculated coefficients for log *J*_MPMO_ fit to the RS equation have the best fit and the best correlation between calculated and experimental log *J*_MPMO_ when utilizing *S*_MO_ as the surrogate for the lipid phase in RS and *S*_7.4_ or S_1.0_ for the aqueous phase in RS for *n* = 8 compounds. Expansion of the dataset is needed to increase the validity of the calculated coefficients. There is a generally poor correlation between the experimental flux and flux calculated using RS equation coefficients derived from previously published experiments using an aqueous or mineral oil donor and human skin *in vitro* or an aqueous donor and silicone membrane*.* The reason that none of the other coefficients to RS worked is because none were obtained using mineral oil as the donor phase and silicone membranes as in the present case.
